# 肺癌基因治疗现状与展望

**DOI:** 10.3779/j.issn.1009-3419.2011.09.11

**Published:** 2011-09-20

**Authors:** 玲玲 祖, 军 陈

**Affiliations:** 300052 天津，天津医科大学总医院，天津市肺癌研究所，天津市肺癌转移与肿瘤微环境实验室 Tianjin Key Laboratory of Lung Cancer Metastasis and Tumor Microenviroment, Tianjin Lung Cancer Institute, Tianjin Medical University General Hospital, Tianjin 300052, China

肺癌是当今发生率和死亡率最高的恶性肿瘤之一，根据病理和临床特征，可以分为小细胞肺癌（small cell lung cancer, SCLC）和非小细胞肺癌（non-small cell lung cancer, NSCLC）两大类^[1]^。其中SCLC约占肺癌发病总数的16.8%，而NSCLC约占80.4%^[2]^。由于肺癌被诊断发现时往往已属晚期，导致患者的5年生存率往往低于10%；随着肺癌诊断和治疗技术的进步，目前肺癌的5年生存率可提高到近15%，但距离理想的治疗效果还有一定的差距^[3]^。常规肺癌治疗多以手术结合放化疗为主，我国还联合中医中药进行综合治疗。由于目前肺癌治疗普遍缺乏针对患者的特异性，往往容易产生较强的毒副作用。近年来，随着肿瘤分子生物学、肿瘤免疫学、生物信息学等学科的迅速发展，使得针对肿瘤的基因诊断、靶向治疗、基因治疗逐渐成为可能，这也就为肺癌的治疗开辟了更加广阔的前景。

基因治疗（gene therapy）是通过分子生物学技术，将目的基因导入患者体内，纠正患者细胞错误的基因表达，从而使疾病得以治疗，是现代医学和分子生物学相结合而诞生的新兴临床医疗技术。基因治疗主要用于治疗危害人类健康的十分严重的疾病，包括遗传病、恶性肿瘤、心血管疾病、感染性疾病等^[4]^。作为疾病治疗的新手段，基因治疗已有一些成功的应用，并逐步向主流医疗发展；基因治疗作为一种特异性强、靶向性好的新型临床治疗方法，越来越被广大的肺癌医学者所重视^[5]^。随着基因治疗技术的发展以及对肺癌发病机制认识的深入，出开展了越来越多的有关肺癌的基因治疗的实验研究甚至临床实验应用。本文将对肺癌的基因治疗的目的原则、治疗方法、载体选择进行综述总结，并展望其可能的前景。

## 1 肺癌基因治疗的目的原则

肺癌的产生是一个多基因参与、逐步发展的复杂过程，其中包括原癌基因的活化、抑癌基因的失活、microRNA表达谱的改变等诸多方面。随着肺癌基础研究的开展，尤其是近年来蛋白质组学、转录本组学、microRNA表达谱的广泛应用，使对肺癌的发生和发展过程的了解更加全面深入。目前已经发现，在肿瘤细胞中基因表达具有一定的特点，例如：凋亡抑制蛋白（inhibotor of apoptosis protein, IAP）过量表达^[6]^、抑癌基因*p53*的突变或缺失^[7]^、血管内皮细胞生长因子（vascular endothelial growth factor, VEGF）表达水平增高^[8]^、microRNA let-7家族表达水平下调^[9]^、microRNA miR-17-92表达增加^[10]^等。基因治疗主要直接针对这些基因表达的变化进行设计，通过分子生物学的手段，抑制癌基因、相关细胞因子或microRNA的过度表达，补偿抑癌基因或相关microRNA的表达，以达到限制肿瘤生长和转移的目的，甚至诱导肿瘤细胞发生细胞凋亡，并诱导免疫系统识别清除肿瘤细胞，最终达到对肿瘤细胞的有效杀伤。肺癌基因治疗的目的原则包括抑制肺癌细胞的生长、抑制肺癌细胞的转移和杀死肺癌细胞。

## 2 肺癌基因治疗方法

围绕上述肺癌基因治疗的目的原则，针对肺癌的发生、发展和转移等各个阶段以及治疗靶位的不同，基因疗法可以概括为以下几种类型：

### 2.1 阻碍原癌基因过量表达治疗

原癌基因的突变激活是肺癌发生的充分条件，原癌基因或其启动子的突变可导致原癌基因的高水平表达，最终导致肺癌细胞的永生化和恶性生长。针对原癌基因的异常活化，设计特异的反义RNA或siRNA进行RNA干扰，可以有效地阻碍原癌基因的过量表达。目前常用的RNA干扰的靶基因包括：*myc*、*neu*、*ras*等。目前已报道，将抑制*c-Myc*基因的miRNA-45表达质粒转染至*ras*基因异常表达的NSCLC细胞中，可以抑制NSCLC细胞的生长^[11]^；*myc*基因的反义RNA体外实验可以抑制SCLC细胞的恶性生长^[12]^。阻碍原癌基因的过量表达治疗，可以抑制肺癌的生长和转移，但不能杀死肿瘤细胞。

### 2.2 补偿抑癌基因表达治疗

补偿抑癌基因表达治疗的方法，是利用分子生物学的技术，将正常的抑癌基因导入肿瘤细胞中，替代原有的突变失活的抑癌基因，或者干扰因抑癌基因的突变造成的信号转导通路的失调，抑制肿瘤细胞的生长和转移，进而诱导肿瘤细胞凋亡。目前常用的抑癌基因包括：*p53*、*p16*、*RB*等；其中又以*p53*基因应用最广，目前已有针对NSCLC治疗携带*p53*基因的重组腺病毒载体的Ⅰ期临床实验^[13]^，相关的治疗效果和毒副作用需进一步的总结。*p53*基因过表达治疗主要针对*p53*基因缺陷的肿瘤细胞，而对无此突变的细胞无效。抑癌基因表达治疗配合放化疗，增强肿瘤细胞敏感性的联合治疗，目前已经进入临床实验阶段^[14, 15]^。

### 2.3 抗血管生成基因治疗

与其它实体肿瘤相同，肺癌是血管依赖性肿瘤，肿瘤血管为肿瘤的生长提供氧气和养分的输送和代谢废物的输出，此外肿瘤血管通常缺乏平滑肌，基底膜有不规则的漏洞，有利于肿瘤的转移。抗血管生成基因治疗主要通过抑制肿瘤血管的生成，抑制肿瘤的生长和转移。目前抗血管生成基因治疗主要包括：针对VEGF及其受体（VEGFR）的基因治疗、血管抑素（vasostatin）基因治疗、内皮抑素（endostatin）基因治疗^[16]^。其中又以VEGF诱导肿瘤血管生成作用最强、特异性最高，为抗血管生成基因治疗的首选^[17]^。

### 2.4 自杀基因治疗

针对肿瘤治疗的自杀基因治疗方法，是通过将表达外源性酶活性的基因导入肿瘤细胞，随后在体内稳定表达的活性酶可以将肿瘤细胞周围的无毒性的药物（称为药物前体）催化成为仅对肿瘤细胞具有细胞毒性的药物，从而达到杀死肿瘤细胞的目的^[18]^。由于在实际应用中，通常采用病毒载体，因此又被称为病毒介导的酶解药物前体疗法（virus directed enzyme prodrug therapy, VDEPT）。自杀基因治疗系统有数十种，其中在肺癌研究中运用最多的是胸腺嘧啶激酶基因/丙氧鸟苷系统（TK/GCV）和胞苷脱氨酶/5-氟胞嘧啶系统（CD/5-FC）。除了针对肿瘤细胞进行治疗，目前已经出现应用TK/GCV系统且以肿瘤血管内皮细胞作为靶细胞进行治疗的尝试^[19]^。但自杀基因治疗仅能杀伤S期细胞，自杀基因的靶向导入问题是该疗法的主要限制因素。

### 2.5 免疫基因治疗

肺癌细胞的免疫原性弱，一般不足以刺激免疫系统对其产生排斥反应^[20]^。免疫基因治疗就是将特定外源基因转入肿瘤细胞中，使其高水平表达相关的细胞因子或MHC抗原，从而激化抗肿瘤的特异性免疫作用。使用遗传工程技术处理肿瘤细胞，使其携带特定细胞因子基因，注射至机体后可以在肿瘤位点持续稳定的表达并释放所携带的细胞因子。注射活的携带特定细胞因子基因的肿瘤细胞，经过增殖会产生更多的抗原并提高细胞因子浓度，直到达到生物学和药理学所需的阈值，引起抗肿瘤的特异性免疫反应。随着免疫反应的进行，肿瘤细胞被杀死，从而关闭了初始的触发作用^[21]^。目前研究常用的细胞因子包括：提高IL-2、INF-γ、TNF-α等从而提高机体免疫系统对肿瘤细胞的识别作用，增强组织相容性抗原等位基因表达，诱导机体的免疫反应。免疫基因治疗具有结合机体免疫的特点，正逐渐成为肺癌基因治疗的研究热点之一^[22]^。

## 3 肺癌基因治疗的载体选择

肺癌基因治疗成功的关键之一在于外源基因在目的细胞中的高效稳定表达，即基因治疗的靶向性、高效性和稳定性，很大程度上取决于基因治疗的载体系统。好的基因治疗载体具备靶向性、无毒副作用、不引起炎症反应和免疫反应等优点。目前所使用的载体系统有十余种，从大的方面可以分为病毒载体和非病毒载体两大类。由于病毒载体较非病毒载体转移效率高，在实际方案中病毒载体占到85%。另一方面，由于病毒载体在安全性、免疫原性方面的局限性，使得越来越多的新兴非病毒载体研究得到发展。下面对肺癌基因治疗的主要载体进行小结。

### 3.1 逆转录病毒载体

逆转录病毒属于RNA病毒家族成员，基因组由两个正义的单链RNA分子组成，被衣壳蛋白包裹。整合酶和逆转录酶结合于衣壳蛋白上。逆转录病毒基因组由两端长末端重复序列、gag、pol、env四部分组成，其中长末端序列起始双链DNA的合成，gag编码衣壳蛋白，pol编码逆转录酶和整合酶，env编码受体识别及衣壳锚定的蛋白。外源目的基因替换*gag*、*pol*、*env*基因后，逆转录病毒不能完成自身复制，但可以将携带的外源目的基因稳定地整合入靶细胞DNA序列中^[23]^。针对这一特性，逆转录病毒可用于作为基因治疗的载体。逆转录病毒载体是目前研究最为深入、应用最早、使用最为广泛的基因治疗载体成员之一。逆转录病毒载体能够非常有效地整合进入靶细胞的基因组中，并持续稳定地表达外源基因，同时具有较高的转导效率。由于逆转录病毒载体只能感染增殖细胞，因此比较适于肿瘤的基因治疗。目前已有使用逆转录病毒载体介导的基因治疗案例，通过抑制肿瘤的血管生成抑制肿瘤的生长^[24]^。但逆转录病毒载体存在特异性低、容量较小、病毒滴度较低、不能满足体积较大肿瘤的治疗要求等缺点，使其在肿瘤基因治疗中存在一定的局限性。近年来新型靶向性逆转录病毒载体及其包装细胞系的构建逐渐克服了逆转录病毒载体特异性低的缺陷^[25]^，使得逆转录病毒载体在肿瘤基因治疗中日趋完善。

### 3.2 腺病毒载体

腺病毒属于双链DNA病毒成员，基因组有36 kb。依据病毒复制中基因表达的时间顺序，腺病毒载体基因组被分成两个主要部分：早期和晚期。早期表达基因包括四个部分，分别命名为E1、E2、E3、E4。晚期表达基因含有五个编码单元，分别是L1、L2、L3、L4和L5。敲除E1后腺病毒无法复制，因此无E1的重组腺病毒载体属于复制缺陷型腺病毒载体^[26]^。外源目的基因整合到载体E1区域可稳定表达。E3和E4的敲除允许大的外源基因插入载体，并减弱病毒的免疫原性。此外，腺病毒载体同时具备其它的特点，包括：相对于其它病毒载体，转导效率较高；可以感染分裂和非分裂的细胞；病毒滴度较高；不整合入染色体，安全性较高；持续表达能力不强且表达周期短。应用于肺癌基因治疗，由于腺病毒感染具有亲肺性，使得腺病毒载体在肺内的转化效率更高，使其成为肺癌基因治疗最常用的病毒载体^[14]^。目前已研制出多种重组的腺病毒载体，克服了传统腺病毒载体易激发机体免疫反应的缺点^[27]^。

### 3.3 腺病毒相关病毒载体

腺病毒相关病毒载体与腺病毒载体的特性极为相似，优点是可以感染分裂和非分裂的细胞且并不引起免疫反应，转导效率较高，安全性好，易于基因操作，易制备高滴度病毒，可利用放射线将病毒从潜伏感染进入裂解感染，为基因治疗联合放疗创造条件；与腺病毒载体不同的是，腺病毒相关病毒载体能够将所携带的外源基因整合入宿主基因组特异的位置，从而可以长时间稳定地表达外源基因。缺点是载体容量有限，可能会出现免疫排斥反应等。随着腺病毒相关病毒新型复制模型的发展，将有更多腺病毒相关病毒载体的基因治疗应用于肺癌治疗^[25]^。

### 3.4 细胞转导肽载体

HIV的Tat蛋白、HSV的VP22蛋白、HBV的前S抗原等蛋白可穿过细胞膜进入细胞，这主要由于该类蛋白中的特殊结构域（一般为10个-30个氨基酸）发挥作用。将这些结构域与其它蛋白、多肽或寡核苷酸等化合物结合或融合表达，可以将生物大分子带入细胞，这些具有携带生物大分子进入细胞的多肽统称为细胞转导肽。目前已成功构建利用Tat转导肽将DNA导入哺乳动物细胞的策略，称为Tat-Phage系统，实验性进行基因治疗^[28]^。

### 3.5 壳聚糖载体

壳聚糖来源于甲壳类动物中甲壳质进行脱乙酰化后得到的多糖物质，属于天然阳离子聚合物。壳聚糖无毒副作用，具有生物粘附性、生物相容性和可降解性。壳聚糖可以与DNA形成稳定的复合物，抵抗血清的降解作用，进入细胞在体内表达报告基因，但基因表达时间较迟^[29]^。壳聚糖作为基因治疗载体的优势为安全无毒，故壳聚糖及其衍生物已经开诊在肺癌中的基础研究。

### 3.6 阳离子脂质体载体

脂质体易于制备，具有较高的生物相容性并可以搭载多种药物，阳离子脂质体表面带有正电荷，易于与带有负电荷的细胞膜相结合，提高转染效率。实验表明，阳离子脂质体通过静脉注射给药、经皮给药以及气管内给药均可以获得较高的转染效率并且明显集中于肺部。阳离子脂质体介导的*mda-7*/*IL-24*基因在体内肺部肿瘤进行实验性基因治疗已经进行^[30]^。

### 3.7 纳米颗粒载体

纳米颗粒载体属于一种新型的聚合体转运系统。纳米颗粒体积小，更易于将携带的目的基因扩散进入细胞。已有基于纳米颗粒载体基因治疗应用于小鼠癌症模型实验证明有较好的抑癌效果。然而，基于纳米颗粒的载体基因治疗可能受限于载体容量，而小容量的纳米颗粒载体可以作为siRNA的有效载体。

## 4 肺癌基因治疗前景展望

肺癌的基因治疗总体上还处于起步阶段，大部分还处于初创或临床Ⅰ期实验阶段，成功应用于临床尚需大量的工作。目前还存在缺少高效传递系统、缺少稳定持续表达以及易产生宿主免疫反应等诸多问题。但有理由相信，随着基础研究的深入，将出现更多的、更为有效的基因治疗靶位和治疗方式；随着大量临床实验的进行，将会出现较为固定和常规的基因治疗模式和方法，成为可供医生选择的治疗手段。由基因治疗自身的靶向性好、可操控等特点，可以预见其将有较好的应用前景。同时随着基因诊断等其它新兴技术的临床应用的普及，将最终实现肺癌患者的基因组水平的个性化诊断。根据诊断结果，将可以实现肺癌的个性化、多靶位的基因治疗，完成对患者手术前肺癌的生长转移控制，术后恢复期的持续治疗最终将大大提高肺癌患者的5年存活率。基因治疗必将成为肺癌治疗的重要工具，最终将造福广大肺癌患者。
